# Human iPSC-Based Models for the Development of Therapeutics Targeting Neurodegenerative Lysosomal Storage Diseases

**DOI:** 10.3389/fmolb.2020.00224

**Published:** 2020-09-18

**Authors:** Marco Luciani, Angela Gritti, Vasco Meneghini

**Affiliations:** San Raffaele Telethon Institute for Gene Therapy (SR-Tiget), IRCCS San Raffaele Scientific Institute, Milan, Italy

**Keywords:** lysosomal storage disorders, central nervous system, induced pluripotent stem cells, neural stem cells, organoids, drug discovery, gene therapy, cell therapy

## Abstract

Lysosomal storage diseases (LSDs) are a group of rare genetic conditions. The absence or deficiency of lysosomal proteins leads to excessive storage of undigested materials and drives secondary pathological mechanisms including autophagy, calcium homeostasis, ER stress, and mitochondrial abnormalities. A large number of LSDs display mild to severe central nervous system (CNS) involvement. Animal disease models and post-mortem tissues partially recapitulate the disease or represent the final stage of CNS pathology, respectively. In the last decades, human models based on induced pluripotent stem cells (hiPSCs) have been extensively applied to investigate LSD pathology in several tissues and organs, including the CNS. Neural stem/progenitor cells (NSCs) derived from patient-specific hiPSCs (hiPS-NSCs) are a promising tool to define the effects of the pathological storage on neurodevelopment, survival and function of neurons and glial cells in neurodegenerative LSDs. Additionally, the development of novel 2D co-culture systems and 3D hiPSC-based models is fostering the investigation of neuron-glia functional and dysfunctional interactions, also contributing to define the role of neurodevelopment and neuroinflammation in the onset and progression of the disease, with important implications in terms of timing and efficacy of treatments. Here, we discuss the advantages and limits of the application of hiPS-NSC-based models in the study and treatment of CNS pathology in different LSDs. Additionally, we review the state-of-the-art and the prospective applications of NSC-based therapy, highlighting the potential exploitation of hiPS-NSCs for gene and cell therapy approaches in the treatment of neurodegenerative LSDs.

## Introduction

Lysosomal storage diseases (LSDs) are a group of inherited genetic disorders caused by the deficiency of lysosomal proteins that leads to the accumulation of undigested storage material. They are rare diseases if considered independently, with a prevalence ranging between 1:57,000 and 1:4,200,000 individuals. However, if taken as a group they are thought to affect up to 1:5,000 live births (Meikle et al., 1999; Poorthuis et al., 1999; Applegarth et al., 2000; Pinto et al., 2004; Poupetova et al., 2010; Al-Jasmi et al., 2013; Platt et al., 2018).

These disorders originate from defects in metabolic enzymes, hydrolases, channels or membrane proteins, cofactors, or components that traffic lysosomal proteins. The accumulation of storage material impairs lysosomal functions and affects several organelles and cellular activities (Ballabio and Gieselmann, 2009). Neurodegeneration is a prominent feature in the majority of LSDs (Wraith, 2002), suggesting an increased sensitivity of neural cells toward dysfunctional cellular clearance.

Enthusiasm has arisen for the possibility to generate neural stem/progenitor cells (NSCs) and differentiated progeny from human induced pluripotent stem cells (hiPSCs), a new source of stem cells obtained by reprogramming of somatic cells through the manipulation of a core set of transcription factors (Ma et al., 2013; Smith et al., 2016). The use of neural cells derived from patient-specific iPSCs to investigate the mechanisms of CNS pathology in these rare disorders has been extensively reviewed (Borger et al., 2017; De Filippis et al., 2017; Zunke and Mazzulli, 2019).

Here, we focus on the advantages and limitations in the use of hiPSC-derived NSCs (hiPS-NSCs) to develop novel therapeutics. In addition, we discuss the prospective application of novel hiPSC-based models to investigate the role of neurodevelopmental defects and to highlight the contribution of non-neural cells and neuroinflammation in the disease onset and progression of neurodegenerative LSDs.

## hiPSC-Derived Neural Populations: Advantages and Drawbacks

Developmental studies in animal models and human embryonic stem cells (hESCs) have increased our knowledge of the key factors regulating the neuralization of pluripotent stem cells, the formation of neuroepithelial cells in neural tube-like structures, and the generation and regionalization of neural stem/progenitor cell populations. The combined inhibition of BMP signaling and Lefty/Activin/TGFβ pathways by co-treatment with Noggin and SB431542 during the first steps of neuroectodermal commitment has significantly increased the efficiency of neuralization from hiPSCs (Chambers et al., 2009). Dual-SMAD inhibition promotes the transient formation of early neuroepithelial cells (NEPs) that spontaneously convert into later-stage NEP populations growing in rosette-like structures (Elkabetz et al., 2008). NEPs can be expanded in presence of FGF2 and EGF for several passages, giving rise to an intermediate developmental stage between rosette-organized NEPs and radial glial cells displaying self-renewal, clonogenicity and responsiveness to instructive cues that promote the induction of distinct neuron subpopulations (Koch et al., 2009; Doerr et al., 2015). Despite displaying self-renewal and multipotency, single-seq RNA-seq analyses revealed the intrinsic heterogeneity hiPSC-derived NEP populations and their enrichment in neuronal and glial progenitors (Lam et al., 2019), with few cell clusters shared with somatic fetal brain-derived human NSCs (hfNSCs) (Lam et al., 2019). Subsequent amplification/selection/enrichment of hiPSC-derived NEPs are needed to obtain radial glial-like populations sharing transcriptional, phenotypic and functional identities with prospectively isolated (CD133^+^ CD34^–^ CD45^–^) hfNSCs (Conti et al., 2005; Elkabetz et al., 2008; Meneghini et al., 2017; Rosati et al., 2018).

Both hiPS-derived NEPs and radial glial cells are generally defined as hiPS-NSCs being characterized by self-renewal, expression of NSC markers (e.g., PAX-6 and Nestin) and multipotency. These features make these cell populations suitable tools for disease modeling studies and the development of novel therapeutic approaches for several neurodegenerative disorders. Indeed, patient-derived hiPS-NSCs can be expanded at medium/large scale and differentiated in neurons, astrocytes and oligodendrocytes, thus recapitulating CNS-specific pathological mechanisms. Importantly, these human *in vitro* models harbor the natural disease-causing mutations in a genetic background which is permissive for the disorder. This feature allows to highlight the role of the genetic background in determining the disease progression and severity, which is particularly relevant in some LSDs (e.g., Metachromatic Leukodystrophy) characterized by high phenotypic variability in patient carrying similar or even identical mutations. Gene knock-out approaches in immortalized cell lines (e.g., SH-SY5Y cell line) cannot fully recapitulate the contribution of the genetic background, also considering that these cellular models are characterized by abnormal karyotypes and the expression of oncogenes that might hamper the identification of disease phenotypes. Additionally, knock-out strategies do not reproduce the many loss-of-function mutations that result in the generation of misfolded proteins potentially leading to LSD secondary pathological mechanisms, including stress pathways activated by the unfolded protein response (UPR) and impaired autophagy. Patient-derived skin fibroblasts harbor the disease-causing mutations and patient-specific genetic backgrounds, and they also display clinical hallmarks in some LSDs, like the accumulation of pathological macromolecules and/or dysfunction in the endosomal–autophagic–lysosomal trafficking (Platt et al., 2012). However, both fibroblasts and highly dividing immortalized cells have different baseline metabolic rates as compared to neural cells. Indeed, they are usually less vulnerable to the toxic effects of substrate accumulation, thus hampering the full investigation of CNS cell type-specific mechanisms.

**FIGURE 1:**
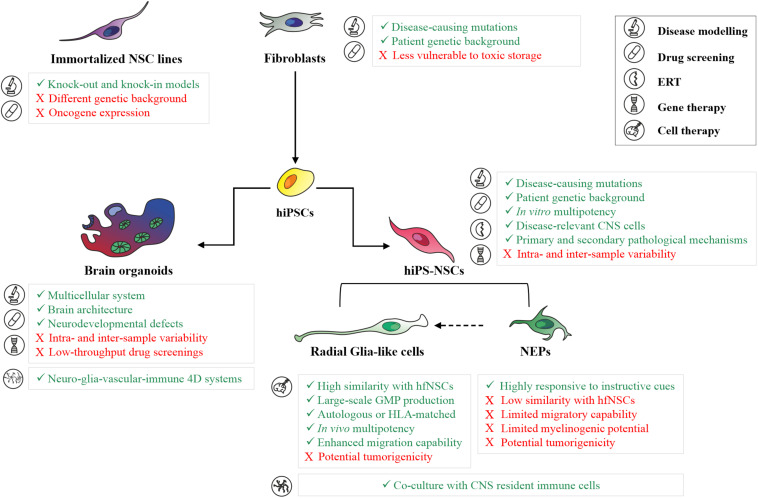
Human 2D and 3D *in vitro* models for basic and translation research in neurodegenerative LSDs. Summary of main advantages (green text) and limitations (red text) in the application of patient skin fibroblasts, immortalized neuronal cell lines, hiPS-NSC cell populations (neuroepithelial cells and radial glial-like cells), and brain organoids for disease modeling studies, development of novel therapeutics (drug screening, enzyme replacement therapy, gene therapy), and cell therapy in neurodegenerative LSDs. ERT, Enzyme Replacement Therapy; NEPs, Neuroepithelial cells.

Despite the recent progress in hiPS-NSC technologies allow the generation of engineered models for high-throughput drug screenings in neurological and psychiatric disease (Hunt et al., 2019), a major challenge in using hiPSC-based models is the potential intra- and inter-experimental variability between donors, clones derived from the same donor and different cell passages of the same clone. To reduce the clone-dependent variability, as well as to dissect the contribution of the genetic background in disease phenotypes, the experimental study might be carefully designed to include isogenic control lines and/or an appropriate number of lines derived from different donors, rather than multiple clones derived from a small number of donors (Germain and Testa, 2017). This is particularly relevant in the study of metabolic processes affected in LSDs (e.g., autophagic flux, lysosomal enzyme activity, and the turnover of macromolecules), which are usually characterized by high baseline variability already in the control cell lines. Considering that a metabolic reprogramming is occurring during neural commitment, in particular in the glycosphingolipid and sulfatide metabolisms (Frati et al., 2018; Russo et al., 2018), quality check-points during the differentiation protocol are critical to define the differentiation stages of patient-specific and control cells. Based on protocols developed in hESCs, the expression of several stage-specific markers can be analyzed to define the hiPSC-derived neural population, including SOX1, N-Cad, Forse-1 expressed in NEP cells, and RC2, SOX2, BLBP, GLAST, CD44 and CD133 expressed in more mature radial glial-like cells (reviewed in Conti and Cattaneo, 2010). The advantages and drawbacks of different 2D *in vitro* models for disease modeling studies and for the development of novel therapeutics are summarized in Figure 1.

## hiPSC-Derived NSCs to Develop New Therapeutic Strategies

Several studies highlighted the relevance of hiPS-NSCs and differentiated neuronal and glial progeny to test the efficacy and safety of therapeutic strategies targeting CNS pathology in LSDs (Table 1). The cell-specificity of LSD pathology has been confirmed in *in vitro* models of Niemann–Pick disease type C (NPC), where several compounds effective in restoring the cholesterol trafficking in patient NPC fibroblasts, including miglustat, suberoylanilide hydroxamic acid, curcumin, lovastatin, pravastatin, and rapamycin (Patterson et al., 2007; Madra and Sturley, 2010; Pipalia et al., 2011), did not display any therapeutic effects in hiPS-NSCs and differentiated progeny (Yu et al., 2014). Additionally, drugs that are effective in both patient’s fibroblasts and hiPSC-derived CNS cells could show a different dose-dependent safety profile. This is the case of the lipid chelators 2-hydroxypropyl-β-cyclodextrin (HPBCD) and 2-hydroxypropyl-γ-cyclodextrin (HPGCD), which favor the degradation/exocytosis of macromolecules through the generation of inclusion complexes with cholesterol, sphingomyelin, lipids and GM2 gangliosides in *in vitro* models of NPC (Soga et al., 2015), Niemann–Pick disease type A (NPA) (Long et al., 2016), Neuronal Ceroid Lipofucinosis (NCL) (Sima et al., 2018) and Tay-Sachs disease (TSD) (Vu et al., 2018). Studies on hiPSC-derived NPC neural cultures showed a dose-dependent neurotoxic effects of HPBCD (Long et al., 2016), which is currently used to treat NPC patients (Matsuo et al., 2013; Ory et al., 2017; Hastings et al., 2019; Ulloa et al., 2020), thus anticipating recent *in vivo* studies showing a region-specific alteration of the homeostasis of different lipid species in the brain of HPBCD-treated mice with potential detrimental effects that should to be carefully evaluated (Long et al., 2016; Glaser et al., 2020).

**TABLE 1:** List of therapeutic strategies applied in hiPSC-derived models to treat primary biochemical defects and secondary pathological dysfunctions in LSD.

Therapeutic strategies	LSDs	Drugs/viral vectors	Cellular models	Therapeutic outcomes	References
Cyclodextrins	NPC1	HPBCD (1 mM) or HPGCD (1 mM)	hiPS-NSC	• Reduction of cholesterol at physiological levels. • Restoration of ATP levels. • Partial rescue of impaired autophagy (p62 clearance).	Soga et al., 2015
		HPBCD (500 μM) or MBCD (300 μM)	hiPS-NSC	• Reduction of cholesterol at physiological level. • Partial restoration of lysosomal trafficking. • Synergistic effects with δ-tocopherol [lower doses of HPBCD (50 μM) or MBCD (20 μM) are required].	Yu et al., 2014
		HPBCD (8 mM) or MBCD (300 μM)	hiPSC-derived neurons	• Reduction of cholesterol accumulation.	Yu et al., 2014
	NPA	HPBCD (1.5 – 6 mM)	hiPS-NSC	• 30–54% reduction of sphingomyelin storage. • Partial restoration of lysosomal trafficking. • Synergistic effects with δ-tocopherol [lower doses of HPBCD (30 μM) are required].	Long et al., 2016
	WD	HPBCD (300 – 600 μM)	hiPS-NSC	• Reduction of accumulation of cholesteryl esters • Partial restoration of lysosomal trafficking.	Aguisanda et al., 2017
	TSD	HPBCD (500 μM)	hiPS-NSC	• 92–97% decrease of GM2 accumulation. • Synergistic effects with δ-tocopherol [lower doses of HPBCD (50 μM) are required].	Vu et al., 2018
	NCL	HPBCD (500 μM – 1 mM)	hiPS-NSC	• Partial recovery of lysosomal trafficking (40–50% decrease of enlarged lysosomes). • Synergistic effects with δ-tocopherol [lower doses of HPBCD (125 μM) are required].	Sima et al., 2018
Tocopherols	NPC1	δ-tocopherol (20 μM)	hiPS-NSC and neurons	• Partial restoration of lysosomal trafficking (in hiPS-NSC). • Reduction of cholesterol accumulation (in hiPS-NSC and neurons).	Yu et al., 2014
	NPA	δ-tocopherol (40 μM) or α-tocopherol (80 μM)	hiPS-NSC	• 30–50% reduction of sphingomyelin storage (highest effects with α-tocopherol). • Partial restoration of lysosomal trafficking.	Long et al., 2016
	WD	δ-tocopherol (5–10 μM)	hiPS-NSC	• Reduction of accumulation of cholesteryl esters. • Partial restoration of lysosomal trafficking.	Aguisanda et al., 2017
	TSD	δ-tocopherol (20 μM)	hiPS-NSC	• 75–83% decrease of GM2 accumulation.	Vu et al., 2018
	NCL	δ-tocopherol (10–40 μM)	hiPS-NSC	• Partial recovery of lysosomal trafficking (10–60% decrease of enlarged lysosomes).	Sima et al., 2018
mTOR-independent enhancers	NPC1	Carbamazepine (100 μM), verapamil (5 μM), trehalose (10 mM)	hiPSC-derived neurons	• Rescue of impaired autophagy (p62 clearance). • Increased survival in neuronal cultures. • No additive effects combining carbamazepine and HPBCD.	Maetzel et al., 2014
Modulator of lipid metabolism and neurogenesis	NPC1	Valproic Acid (1 mM)	NSC derived from direct reprogramming of fibroblasts	• Activation of LXR β pathway. • Reduction of cholesterol at physiological levels. • Partial restoration of lysosomal trafficking. • Recovery of self-renewal NSC potential.	Sung et al., 2017
Modulators of calcium and WNT signals	NPC1	Curcumin (10 μM), dantrolene (10 μM) or BIO (10 μM)	hiPSC-derived neurons	• Increased neuronal viability.	Hsieh et al., 2004
Regulators of inflammasome	GM1	Z-YVAD-FMK (10 μM) or IL1RA (1 mg/ml)	hiPS-NSC	• Downregulated expression of inflammasome factors. • Recovery of morphological abnormalities in neurospheres. • Reduced release of pro-inflammatory cytokines upon hiPS-NSC transplantation.	Son et al., 2015
ERT	NPA	Human ASM (187.5 nM)	hiPS-NSC	• Partial reduction of sphingomyelin storage.	Long et al., 2016
	WD	rhLAL (0.3 – 2.7 μM)	hiPS-NSC	• Reduction of accumulation of cholesteryl esters. • Partial restoration of lysosomal trafficking.	Aguisanda et al., 2017
	TSD	rhHEXA (100 nM)	hiPS-NSC	• Strong reduction of GM2 accumulation at physiological levels.	Vu et al., 2018
	NCL	rhPPT1 (200 nM) or rhTPP1 (200 nM)	hiPS-NSC	• Restoration of lysosomal trafficking at physiological levels in hiPS-NSC derived from infantile (CLN1/PPT1) and late infantile (CLN2/TPP1) NCL patients.	Sima et al., 2018
Gene Therapy	MPS IIIC	LV.pCMV.hHGSNAT	hiPSC-derived neurons	• Supraphysiological (50–150 fold higher) enzymatic activity. • Recovery of network connectivity in mature neurons.	Canals et al., 2015
	MLD	LV.pPGK.hARSA or bdLV.pPGK.hARSA.GFP	hiPSC-derived NSC, neurons, and glial cells	• Supraphysiological (5–40 fold higher) enzymatic activity, highest in differentiated cultures. • Recovery of sulfatide storage and composition in hiPS-NSC and differentiated progeny. • In hiPS-NSC: recovery of lysosomal trafficking and NSC differentiation potential; • In hiPS-NSC differentiated cultures: rescue of cellular stress and apoptosis.	Frati et al., 2018
	NCL	AAVrh10.pCAG.hCLN2 or AAVrh10.pCAG.hCLN3	hiPS-NSC	• Reduced subunit c storage at physiological levels.	Lojewski et al., 2014
	GM1	AAVrh9.pCAG.hGLB1	Cerebral organoids	• 50% of physiological enzymatic activity. • Reduction (50–75%) of GM1 storage.	Latour et al., 2019

*NPC1, Niemann–Pick disease type C 1; NPA, Niemann–Pick disease type A; WD, Wolman disease; TSD, Tay-Sachs disease; NCL, Neuronal Ceroid Lipofucinosis; GM1, GM1 ganglosidosis; MPS, Mucopolysaccharidosis; MLD, Metachromatic Leukodistrophy; HPBCD, 2-hydroxypropyl-β-cyclodextrin; HPGCD, 2-hydroxypropyl-δ-cyclodextrin; MBCD, methyl-β-cyclodextrin; LXR β, Liver X receptor β; ASM, Acid Sphingomyelinase; LAL, Lysosomal Acid Lipase; HEXA, Hexosaminidase A; PPT1, Palmitoyl-protein thioesterase 1; TTP1, Tripeptidyl-peptidase 1; HGSNAT, Heparan-α-glucosaminide N-acetyltransferase; ARSA, Arylsufatase A; CLN, ceroid lipofuscinosis; GLB1, β-galactosidase 1; pCMV, Cytomegalovirus promoter; pPGK, human Phosphoglycerate Kinase promoter; pCAG, composite of the CMV early enhancer and chicken beta-actin promoter.*

Human iPS-NSCs can be exploited to define novel therapeutic strategies counteracting substrate accumulation in affected lysosomes. For example, studies on hiPS-NSCs proposed a complementary approach based on tocopherols to stimulate the exocytosis of macromolecules *via* the phagocytic, caveolae-, clathrin-, and cell adhesion molecule (CAM)-mediated pathways to restore the endosomal-lysosomal trafficking (Xu et al., 2012; Manthe et al., 2019). Both δ- and α-tocopherol significantly reduced the storage in NPC and NPA hiPSC-derived NPCs and neurons (Yu et al., 2014; Long et al., 2016), in agreement with previous observations showing that α-tocopherol-rich diet increases the survival of Purkinje neurons, reduces astrogliosis, and improves coordination and locomotor functions in NPC mouse models (Marin et al., 2014). Importantly, the use of hiPS-NSCs demonstrated that δ-tocopherol acted in synergy with low HPBCD doses, thus paving the way to future *in vivo* studies aimed at demonstrating that the co-administration of these drugs could be a safer and more effective therapeutic option for the treatment of several LSDs, including NPC, NPA, TSD and NCL (Yu et al., 2014; Long et al., 2016; Sima et al., 2018; Vu et al., 2018). These studies highlight that data collected in hiPSC-derived neural cells are not only accessory to *in vivo* studies in disease animal models, but could provide deeper insights on the metabolism, dose-response effects and toxicity of drugs designed for the treatment of LSDs.

The capability of hiPSC-derived neural cultures to recapitulate not only the primary biochemical defects but also secondary pathological mechanisms, stress the relevance of these cellular models in drug discovery processes aimed at identifying novel drugable pathways targeting common pathological mechanisms shared by several LSDs. Human iPSC-derived neurons from Gaucher disease (GD) patients show defects in the clearance of autophagosomes responsible of impaired calcium homeostasis and cytotoxicity (Schondorf et al., 2014; Awad et al., 2015). Similarly, the autophagic flux is impaired in NPC hiPS-NSCs and neurons (Trilck et al., 2013; Lee et al., 2014a; Maetzel et al., 2014; Soga et al., 2015; Trilck et al., 2017). Interestingly, mTOR-independent autophagy enhancers (Table 1) are cytoprotective (Maetzel et al., 2014). Also, recent studies suggest the contribution of Transcription Factor EB (TFEB) (Awad et al., 2015; Napolitano and Ballabio, 2016; Puertollano et al., 2018) and/or the VEGF/VEGFR2-mediated sphingosine kinase (SphK) activity (Lee et al., 2014a) to the impaired autophagy in hiPSC-derived neurons. In macrophages derived from peripheral monocytes of patients affected by type 1 Gaucher disease, inflammasome activation has been linked to impaired autophagy and increased IL-1β secretion (Aflaki et al., 2016). Similarly, accumulation of gangliosides, as well as other pathological substrates, leads to lysosomal disruption, inflammasome activation and altered autophagic flux responsible of reduced self-renewal potential and morphological abnormalities in hiPS-NSCs (Son et al., 2015). Upregulation of inflammatory caspases (e.g., CASP1) and IL-1β pathway are rescued by treatment with the CASP1 inhibitor Z-YVAD-FMK and the natural competitive IL1R antagonist Anakinra (Son et al., 2015) (Table 1). Despite the molecular mechanisms responsible of impaired autophagy in LSDs are still under investigation, autophagy is emerging as a relevant mechanisms involved in the clearance of pathological substrates, lysosomal trafficking and inflammation in different LSDs with important implications for the development of new therapeutic approaches (Lee et al., 2014a; Maetzel et al., 2014; Soga et al., 2015; Rabenstein et al., 2017; Seranova et al., 2017; Bayo-Puxan et al., 2018; Swaroop et al., 2018; Teinert et al., 2020).

Beside drug discovery, hiPS-NSCs have been exploited to investigate the efficacy and safety of therapies aimed at restoring the expression of the deficient lysosomal enzyme in disease-target cells (Table 1). The efficient uptake of recombinant human enzymes in enzyme replacement therapy (ERT) settings has been demonstrated in hiPSC-derived CNS cells in NPA, infantile and late infantile NCL, TSD and WD (Long et al., 2016; Aguisanda et al., 2017; Sima et al., 2018; Vu et al., 2018). Lentiviral vector (LV)-mediated overexpression of ARSA or heparan-α-glucosaminide *N*-acetyltransferase (HGSNAT) in MLD and MPSIIIC hiPS-NSCs, respectively, rescued both the primary storage and neurodevelopmental defects, without cytotoxic effects induced by transgene overexpression (Canals et al., 2015; Frati et al., 2018). These *in vitro* studies in relevant human models could provide deeper insights on the regulation of cross-correction mechanisms in neural cells, and on dose-dependent cytotoxicity of the delivered/over-expressed enzyme and genotoxicity of viral vectors in a human setting, which are relevant in perspective of the clinical translation of these approaches. In this view, they complement *in vivo* studies in small and large animal models, which are, however, indispensable to define the distribution of genetically modified cells and/or viral particles in GT approaches and the kinetics of enzyme distribution in both ERT and GT approaches (Lattanzi et al., 2010, 2014; Meneghini et al., 2016; Gigliobianco et al., 2019; Ohashi, 2019).

## Novel iPSC-Models to Advance Basic and Translational Research

Patient-specific NSCs are relevant models to address the neurodevelopmental defects that could anticipate the disease onset and/or exacerbate disease progression in LSD patients (Figure 1). Progressive accumulation of undegraded substrates, alteration of endosomal-lysosomal and autophagic pathways, defective calcium homeostasis, and endoplasmic reticulum (ER)-mediated stress have been associated with impaired neural commitment, reduced self-renewal and differentiation potential, altered astroglial-neuronal interactions and synaptic transmission in hiPS-NSCs and differentiated progeny derived from LSD patients (Lojewski et al., 2014; Canals et al., 2015; Son et al., 2015; Bayo-Puxan et al., 2018; Frati et al., 2018; Kobolak et al., 2019; Matsushita et al., 2019). These neurodevelopmental defects are only partially recapitulated by LSD mouse models, due to the less vulnerability to substrate accumulations during fetal brain development and/or the presence of compensatory mechanisms decreasing the neurotoxic effects.

Studies on hiPS-NSCs suggested that treatments counteracting primary and secondary biochemical defects also exert independent pro-neurogenic effects (Table 1). Besides the effects of valproic acid (VPA) on Liver X receptor (LXR) β-dependent cholesterol metabolism (Sung et al., 2017) and on the folding and trafficking of mutated NPC1 protein (Subramanian et al., 2020), the pro-neurogenic effects of VPA (Hsieh et al., 2004; Yu et al., 2009; Vukicevic et al., 2015) are potentially contributing to rescue the defective self-renewal and neurogenic potential in NPC NSCs (Sung et al., 2017). Additionally, premature cell death in NPC neurons caused by impaired cholesterol homeostasis is rescued by restoring NSC biological processes regulated by calcium and WNT pathways through treatment with GSK3- or calcium-inhibitors (Hsieh et al., 2004; Efthymiou et al., 2015).

Most of these studies have been performed using 2D *in vitro* cultures, either enriched in a specific cell type or using mixed differentiated populations. While this is advantageous in defining cell-autonomous pathological mechanisms or neurodevelopmental defects affecting specific neuronal and/or glial subpopulations, this system does not faithfully mimic the high degree of morphological and functional complexity of the human brain. Recent advances in stem cell technologies may address and overcome these limitations through the generation of iPSC-based self-assembled 3D cultures (brain/cerebral organoids) composed of neural stem/progenitor cells, neurons and glial cells organized in tissue-like structures recapitulating relevant steps of the human fetal brain development, including the spatiotemporal dynamicity of neurogenesis, the formation of regional neural circuitries, and the integration of glial cells into neural networks (Di Lullo and Kriegstein, 2017; Quadrato and Arlotta, 2017). The first protocols developed to generate brain/cerebral organoids relied on the spontaneous capability of hiPSCs to differentiate in self-organized neuroepithelial structures giving rise to different brain regions (e.g., forebrain, midbrain and hindbrain, retina, choroid plexus) (Lancaster and Knoblich, 2014). The few studies reporting early neurodevelopmental defects in hiPSC-derived cerebral organoids of LSD patients are based on this “spontaneous” and “unguided” method. The use of this system in association to OMIC techniques (e.g., whole-transcriptome and metabolomics analyses) allowed the identification of early disease-specific signatures affecting CNS development, corticogenesis, synaptogenesis and neurotransmission in NCL-derived brain organoids, suggesting that disease-associated changes occur before disease onset (Gomez-Giro et al., 2019). 3D organoid models derived from Hexosaminidase B (HEXB) deficient hiPSCs or GLB1 knockout hiPSCs showed a progressive accumulation of GM2 and GM1 gangliosides during neurodevelopment (Allende et al., 2018; Latour et al., 2019). In particular, organoids derived from Sandhoff patients showed not only an impairment in pathways regulating CNS development and neuronal differentiation, but also increased cellular proliferation and organoid size (Allende et al., 2018). Interestingly, these human 3D brain models were successfully used to evaluate the efficacy of AAV-mediated GT (Table 1) (Allende et al., 2018; Latour et al., 2019), being not only a suitable model to investigate neurodevelopmental defects in a complex multicellular system mimicking the human brain architecture, but also a potential intermediate step between animal models and patients to evaluate vector tropism in a multicellular complex system mimicking human brain (Figure 1).

Despite the relevance of data collected in these studies, single-cell transcriptomic studies show that organoids generated using these protocols (from the same hiPSC lines or different donors) are characterized by high variability in cell and brain-region composition (Kanton et al., 2019). The recent development of “guided” methods based on small molecules and growth factors instructing the hiPSC differentiation processes allowed the generation cerebral organoids with reduced intra- and inter-sample variability (Qian et al., 2019; Velasco et al., 2019). These protocols are more suitable for disease modeling studies, in particular in the investigation of the dynamic metabolic processes affected in LSDs. Future studies combining the use of these advanced 3D models generated from engineered isogenic lines and single cell technologies could dissect the contribution of different cell subpopulations in neurodevelopmental defects and neurodegenerative mechanisms affecting LSD brains.

The majority of current 2D and 3D hiPSC-based models are designed to investigate pathological mechanisms in neurons. However, growing evidences show an important contribution of glial cells in LSD pathology. Indeed, the survival of astrocytes in 2D culture models of Hunter Syndrome is affected by the depletion of ER luminal Ca^2+^ storage and the increased cytoplasmic ion concentration induced by the accumulation of glycosaminoglycans (GAGs) during hiPS-NSC differentiation (Kobolak et al., 2019). Additionally, reduced protein kinase C-mediated phosphorylation of the intermediate filaments vimentin and GFAP leads to an astrogliosis-like phenotype in hiPSC-derived NPC astrocytes, suggesting that gliosis is a primary effect of the cholesterol accumulation within glial cells and not a secondary mechanism mediated by the neuronal loss in NPC (Peter et al., 2017). Finally, ARSA deficiency leads to impaired commitment/maturation of astrocytes and oligodendrocytes besides neurons in Metachromatic Leukodystrophy (MLD) (Frati et al., 2018). These data highlight the potential contribution of altered gliogenesis, astrogliosis and dysfunctional glia-neuronal interactions in LSD pathology which might precede or be concomitant to the appearance of pathological signs in the neuronal compartments. The development of protocols favoring the generation of astrocytes and mature myelinating oligodendrocytes in cerebral organoids (Madhavan et al., 2018; Marton et al., 2019) will improve the study of the neuron-glia interactions in neurodegenerative processes (Frati et al., 2018; Kobolak et al., 2019) and the definition of pathological mechanisms regulating oligodendrocyte degeneration in demyelinating LSDs (Frati et al., 2018).

Additionally, microglia and infiltrating macrophages are important players in the pathology and treatment of LSDs. Recent studies in GD, NPC1 and Sandhoff disease mice suggested that accumulation of macromolecules in the lysosomes of neurons results either directly or indirectly in the activation of neighboring microglia which generate a neuroinflammatory response contributing to the propagation of neuronal damage in affected brains (Cho et al., 2019). On the contrary, the role of infiltrating monocyte-derived macrophages is still controversial, with reports showing their contribution to the pathology in Sandhoff mice (Wu and Proia, 2004; Kyrkanides et al., 2008) and a recent study demonstrating the absence of infiltrating macrophages in the brain of GD, NPC1 and Sandhoff mice. In GT settings based on autologous transplantation of LV engineered hematopoietic stem cells, busulfan treatment induces myeloablation and the permeabilization of blood-brain barrier, thus favoring microglia reconstitution and infiltration of macrophages derived from myeloid cells over-expressing the lacking enzyme, which enable enzymatic cross-correction of deficient CNS cells (Biffi et al., 2013; Sessa et al., 2016). Therefore, human LSD models based on 3D human tri- and tetra-culture and brain-on-a-chip platforms integrating neuro-glia-vascular-immune system (Sharma et al., 2020) could be intriguing models to dissect the contribution of microglia and infiltrating macrophages in disease onset and progression, and optimize the molecular mechanisms regulating enzymatic cross-correction mediated by myeloid cells.

## hiPSC-Derived Nscs as a Valuable Cell Source for Cell and Gene Therapy Approaches

Since pioneer studies in rodent and human NSCs (Reynolds et al., 1992; Gritti et al., 1996; Vescovi et al., 1999), advances in methodology and protocols have allowed to efficiently isolate and expand somatic NSCs from fetal and adult tissues (Conti and Cattaneo, 2010) while preserving their self-renewal and multipotency capabilities. These cells represent an important source to test innovative cell-based approaches in pre-clinical studies and, ultimately, in patients.

Pre-clinical studies in animal models demonstrated efficient NSC engraftment, migration toward the damage areas and persistence in the perivascular “niches” upon intracerebral transplantation in neonatal and adult rodents (Martino and Pluchino, 2006; Bacigaluppi et al., 2009) and non-human primates (Pluchino et al., 2009; Kutikov et al., 2019). Increased evidences suggest that neurodegenerative disorders are associated with multicellular dysfunctions (Olivera-Bravo et al., 2016; Gleichman and Carmichael, 2020; Greenhalgh et al., 2020; Rodriguez-Gomez et al., 2020) that might be only partially rescued by transplantation of committed neuronal and glial progenitors or specific neuronal subpopulations. The “bystander effects” mediated by neurotrophic, neuroprotective, and immunomodulatory factors released by NSCs (Park et al., 2002; Lu et al., 2003; Pluchino et al., 2005; Richardson et al., 2005), and the NSC potentiality for cell replacement through the generation of both neuronal and glial progeny upon transplantation (Lee et al., 2014b) might potentially show improved clinical outcomes as compared to transplantation of committed progenitors or neurons. These features have been exploited for the treatment of acute and chronic neurodegenerative disorders, such as stroke (Bacigaluppi et al., 2016), Alzheimer disease (Ager et al., 2015), Parkinson disease (PD) (Lee et al., 2014b), Amyotrophic Lateral Sclerosis (ALS) (Xu et al., 2006; Park et al., 2009), and Multiple Sclerosis (MS) (Pluchino et al., 2009).

The development of consistent GMP-scale procedures for NSC isolation/enrichment (i.e., CD133 membrane-bound sorting) (Uchida et al., 2000), expansion and cryopreservation (Crook and Tomaskovic-Crook, 2017) paved the way to the clinical translation of NSC-based cell therapies for the treatment of neurodegenerative disorders. Phases I/II clinical trials based on the transplantation of human NSCs of fetal origin (human fetal NSCs, hfNSCs) have been authorized to treat ALS (NTC01640067; Mazzini et al., 2015), MS (NCT03282760, NCT03269071), PD (NCT03128450), Cerebral Palsy (NCT03005249), Ischemic Stroke (NCT03296618), Spinal cord injury (NCT02163876, NCT01725880, NCT01321333), infantile and late infantile Neuronal Ceroid Lipofuscinosis (Selden et al., 2013, NCT00337636, NCT01238315), and Pelizaeus–Merzbacher disease (NCT01005004, NCT01391637). These clinical trials documented the tolerability of the surgical procedures, the engraftment and persistence of transplanted NSCs and some evidence of stabilization of the clinical phenotypes (Selden et al., 2013; Mazzini et al., 2015; Tang et al., 2017). Despite these promising results, hfNSC-based cell therapy presents some drawbacks, including the requirement of a high number of donor cells that could be difficult to expand in large-scale GMP production, and immunosuppressive regiments to avoid the immune rejection of transplanted cells and graft-vs-host disease.

Some of these caveats might be overcome with the use of pluripotent-derived NSC. In fact, pluripotent stem cells could be: (i) isolated from human embryos (e.g., embryonic stem cells, ESC) or generated through highly efficient transgene-free reprogramming (e.g., hiPSC) from easily accessible cell sources (Haase et al., 2017); (ii) easily genetically modified through efficient and safe LV-mediated gene addition strategies (Doerr et al., 2015; Vanhee and Vandekerckhove, 2016; Meneghini et al., 2017) or site-specific genome-editing (Merkert et al., 2017); (iii) clonally expanded and differentiated for large-scale GMP production of transplantable cells (Zweigerdt et al., 2011) (Figure 1).

Several pluripotent stem cell-based clinical trials are ongoing or recruiting patients (Martin, 2017). ESCs-based therapies have been proposed for the treatment of PD (NCT03119636), ALS (NCT03482050) or Dry Age-related Macular Degeneration (NCT03046407, NCT03167203, NCT02590692). The prospective clinical application of iPSC-derived cells can overcome ethical and immunological issues related to the transplantation of ESC-derived cells. Retinal pigmented epithelium (RPE) cells derived from both autologous and human leukocyte antigen (HLA)-matched allogeneic hiPSCs are currently applied for the treatment of Macular Degeneration patients in Japan (Mandai et al., 2017) and in the United States. In 2018, a patient affected by PD has been transplanted with 2.4 million dopaminergic neuronal progenitors derived from allogenic hiPSC (Cyranoski, 2018) and a first clinical trial based on hiPS-NSCs started in 2019 (NCT03815071).

Prospective NSC-based cell therapy approaches for LSDs have to face an additional layer of complication, i.e., the coupling with GT strategies to provide the functional protein in the affected cells. In addition, pre-clinical studies and clinical observations highlight the need of supraphysiological expression of the deficient enzyme in transplanted cells to achieve therapeutic levels of functional enzyme in affected tissues, specifically in CNS (Gritti, 2011; Biffi, 2016; Penati et al., 2017) (Table 2). Human iPS-NSC-based gene and cell therapies could be exploited for the treatment of LSDs with prevalent CNS pathology [e.g., Batten disease (Jankowiak et al., 2015), and GM2 gangliosidoses (Lacorazza et al., 1996)] or in combined strategies with autologous transplantation of enzyme-overexpressing hematopoietic stem cells (HSC) to treat LSDs with central and peripheral neuropathies and affected visceral organs, i.e., GLD (Ricca et al., 2015).

**TABLE 2:** Summary of pre-clinical cell therapy approaches based on human and murine neural stem/progenitor cells for the treatment of neurodegenerative LSDs.

LSDs	Transplanted cell source	Gene therapy approach	Injection site	Therapeutic outcomes	References
MLD	hiPS-NSCs (hiPSC-derived radial glia-like cells)	Lentiviral vectors bdLV.pPGK.hARSA.GFP and LV.pPGK.hARSA	Corpus Callosum (PND60 mice – unilateral injection) or lateral ventricles (PND1 mice – bilateral injection)	• 70% of ARSA physiological activity in CNS regions up to 6 months post-transplantation; • Decrease of sulfatide storage in white matter regions.	Meneghini et al., 2017
	hiPSC-derived NEPs and APCs	Lentiviral vector pLentiWE-pEF1α.hARSA-IRES-eGFP	Lateral ventricle (PND1 mice – unilateral injection)	• Decrease of sulfatide storage up to 300 μm from injection site.	Doerr et al., 2015
	mNPCs		Lateral ventricle (PND2 mice – bilateral injection)	• 47% of ARSA physiological activity in the cortex; • 50–70% reduction of sulfatide deposits and decreased neuronal degeneration; • Improved locomotor performances.	Givogri et al., 2008
	mNPCs	Lentiviral vector pHIV-CS-pCAG.ASA-PRE(ppt)	Hippocampus	• 70% reduction of sulfatide deposits.	Kawabata et al., 2006
GLD	Combined transplantation of human bone marrow cells and engineered mNPCs	Lentiviral vector bdLV.pPGK.GALC.eGFP	Lateral ventricles (PND2 mice – bilateral injection of engineered mNSCs)	• 30–40% of physiological GALC activity; • Storage clearance (50–70%); • Delayed astrogliosis, improved myelination and reduction of neuronal loss; • Enhanced preservation of myelinated axons in the sciatic nerves; • Enhanced lifespan of treated mice.	Ricca et al., 2015
	mNPCs or hfNSC	Lentiviral vector bdLV.pPGK.GALC-HA.GFP	Lateral ventricles (PND2 mice – bilateral injection)	• Reduced tissue storage (30-50%); • Decreased neuroinflammation; • Delayed onset of neurological symptoms and longer lifespan.	Neri et al., 2011
	Transformed mNPCs (MAR-52)	Retroviral vector MFG.mGALC	Central hemispheres (PND2 mice – bilateral injection)	• Reduced astrogliosis, recovery of oligodendrocyte morphology and remyelination; • Modest increase in lifespan and body weight.	Strazza et al., 2009
MPS IIIB	Murine iPS-NSCs	Lentiviral vector SAM.NAGLU.GFP	Lateral ventricle (PND0 and PND1 mice – bilateral injection)	• 1.3-fold decrease of storage material; • Reduction in astrocyte activation; • Prevention of microglial activation.	Clarke et al., 2018
MPS VII	hiPS-NSCs	PiggyBac vector pCAG.GUSB	Striatum (PND60 mice – bilateral injection)	• Recovery of microglia-mediated inflammation.	Griffin et al., 2015
	HB1 human NSC line	Retroviral vector pLHCHBG	Lateral ventricle (PND0 and PND1 mice)	• supraphysiological expression of human β-Glucuronidase (GUSB); • Clearance of lysosomal GAG storage.	Meng et al., 2003
	mNPCs C17.2		Lateral ventricle (neonatal mice)	• GUSB expression (38% of physiological level) up to 3 weeks post-transplantation; • Widespread clearance of lysosomal storage.	Snyder et al., 1995
	hNPCs	Lentiviral vector pTRIPΔU3.CMVp.GUS	Left and right striatum (adult mice)	• Transgene expression up to 6 months after transplantation.	Buchet et al., 2002
Sandhoff disease	mNPCs C17.2		Hippocampus (PND90 mice – unilateral injection)	• HEX reconstitution (6% of WT level); • 19% reduction in GM2 storage; • Decreased inflammation; • Delayed disease progression and increased lifespan.	Jeyakumar et al., 2009
TSD	mNPCs C17.2 or C27.3 lines	Retroviral vector BPHαSH	Lateral ventricles (fetal E14.5 and neonatal mice)	• Up to 43% of HEXA physiological activity.	Lacorazza et al., 1996
NPA	mNPCs C17.2 or hNPCs HFT13		Lateral ventricles and cerebellum (PND1 and PND3 mice)	• Decrease in cholesterol accumulation; • Decrease in neuronal and glial vacuolation; • No improvements in Purkinje neurons loss, but improved rotarod performance.	Sidman et al., 2007
	mNPCs	Retroviral vector	Hippocampus and thalamus (PND4/5 mice), or hippocampus, thalamus, and striatum (5, 8, and 28 week-old mice)	• Low hASM detection; • Reversal of distended lysosomal pathology; • Regional clearance of sphingomyelin and cholesterol.	Shihabuddin et al., 2004
NPC1	mNPCs C17.2		Cerebellum (PND1 mice)	• Increased lifespan lengthening.	Ahmad et al., 2007
NCL	mNPCs-CTNF	Lentiviral vector pCAG-CNTF-IRES-Venus-2A-ZEO	Intravitreal transplantation (PND14 mice)	• Attenuated photoreceptor loss.	Jankowiak et al., 2015

*MLD, Metachromatic Leukodistrophy; GLD, Globoid Cell Leukodystrophy; MPS, Mucopolysaccharidosis; TSD, Tay-Sachs disease; NPA, Niemann–Pick disease type A; NPC1, Niemann–Pick disease type C 1; NCL, Neuronal Ceroid Lipofucinosis; mNPCs, murine Neural Progenitor Cells; NEPs, Neuroepithelial cells; APC, Astroglial Progenitor Cells; hfNSC, human fetal Neural Stem Cells; MAR-52, spontaneously transformed murine progenitor cell line; HB1 human NSC line, immortalized human NSC clone; mNPCs C17.2 or C27.3, immortalized mouse neural progenitor cell line; hNPCs HFT13, human neural precursor cell line derived from human fetal week 13 telencephalon; mNPCs-CTNF, murine Neural Stem Cell line overexpressing CTNF; PND, postnatal day; pPGK, human Phosphoglycerate Kinase promoter; pEF1alpha, human Elongation Factor-1 alpha promoter; pCAG, composite of the CMV early enhancer and chicken beta-actin promoter; pCMV, Cytomegalovirus promoter.*

A proof-of-principle of the potential application of hiPS-NSCs in autologous *ex vivo* GT protocols, have been recently provided in mouse models of Sly disease and MLD. Bilateral striatal injections of hiPS-derived neural stem/progenitor cells overexpressing β-glucuronidase reduced neuroinflammation and microglia activation in the surrounding tissues, already at 4 weeks post-transplantation, suggesting that cross-correction mechanisms and bystander effects can cooperate in reducing neuroinflammation in LSD affected brains (Griffin et al., 2015). Intracerebral transplantation of LV-transduced MLD hiPS-NSC expressing supraphysiological ARSA levels into neonatal and adult immunodeficient MLD mice provided robust and long-lasting enzymatic supply in the whole CNS (Meneghini et al., 2017). Of note, reduction of the pathological sulfatide storage has been achieved also by transplanting hiPS-NSCs with a relatively low number of copies of integrated vector, due to their capabilities of repopulating the entire brain, in particular in neonatal settings, and of secreting therapeutically relevant levels of enzyme in the cerebrospinal fluid, thus inducing cross-correction in CNS regions far from the injection sites (Meneghini et al., 2017).

The stage of neural commitment of transplanted cells seems to be particularly relevant in cell therapy approaches because of its impact on their multipotent and migration capabilities. In the context of widespread demyelinating disorders, like MLD, transplantation of ARSA-overexpressing neuroepithelial (NEP) cells, with limited migration and myelinogenic potentials resulted in reduction of the sulfatide storage only in regions closed to the injection sites (Doerr et al., 2015). Conversely, hiPS-NSC sharing molecular, phenotypic and functional identity with human fetal NSCs showed an enhanced rostro-caudal distribution upon transplantation, in particular in the white matter areas, and the capability to differentiate in myelinating oligodendrocytes, thus potentially combining multiple therapeutic effects, i.e., increased production of ARSA from glial cells, widespread distribution of enzyme along CNS, and cell replacement (Meneghini et al., 2017).

Despite the justified enthusiasm, hiPS-NSC-based approaches have to face several challenges before reaching full clinical exploitation. The production of patient-specific hiPS-NSCs for personalized medicine is costly and time-consuming. The generation of HLA-typed hiPSC banks derived from healthy donors (50–150 HLA-typed donors could cover almost the 90% of the United Kingdom and Japan populations, Taylor et al., 2012) could overcome these limitations, favoring the broad application of hiPS-NSC-based cell therapy for the treatment of neurodegenerative disorders, including diseases characterized by a rapid progression, such as LSDs.

A major concern related to the application of hiPS-NSCs is their safety profile. Indeed, the potential spontaneous reactivation of master regulators of pluripotency in engrafted cells (Choi et al., 2014; Nori et al., 2015) or the transplantation of partially differentiated cells could give rise to the appearance of brain cancers or disrupt pre-existing neuronal circuits. In this perspective, the recent development of hiPSC lines with drug-inducible suicide genes (Caspase9-FKBP^F36V^ fusion protein or herpes simplex virus-derived thymidine kinase) inserted immediately downstream to hiPSC-specific genes (e.g., *NANOG*) or constitutively expressed genes (e.g., *ACTB*) could provide methods to deplete teratoma-forming cells *prior* to transplantation or eliminate the entire populations of engrafted cells in presence of adverse events (Martin et al., 2020). To increase the quality of the transplanted hiPSC-based cell products, the validation of the transcriptional and epigenetic landscapes of hiPS-NSCs in comparison to clinically relevant human fetal NSCs could be important to ensure the silencing of genes and enhancers associated with pluripotency and tumorigenicity and to verify the maturation stages of transplanted cell populations. Additionally, single cell analyses of hiPS-NSC populations aimed to identify membrane-bound cell-specific markers could implement the prospective isolation of “pure” hiPS-NSCs or neuronal and glial progenitors with peculiar features (i.e., anti-inflammatory and neuroprotective effects in NSCs, and enhanced neurogenic or gliogenic potential in progenitors) that could be exploited to improve the recovery of disease-specific damages in neurodegenerative LSDs. The application of novel multi-omics technologies and editing platforms optimizing the production of hiPS-NSCs with a “*bona fide* neural transcriptional signature” coupled to *in vitro* and *in vivo* functional studies seems to be mandatory to make reliable the translation of hiPS-NSC transplantation in clinics.

## Conclusion and Perspective

Several neurodegenerative LSDs have been modeled by using hiPSC-derived NSCs and differentiated progeny, allowing the definition of cell type-specific pathological pathways downstream the primary biochemical defects (Borger et al., 2017; De Filippis et al., 2017; Zunke and Mazzulli, 2019). Early neurodevelopmental defects and impaired cellular crosstalk within the neuro-glia and neuro-immune systems seem to play a crucial role in the onset and development of CNS pathology in several LSDs. The collection of patient-specific hiPSCs in cell banks, the standardization of neural differentiation protocols, and the exploitation of novel 2D and 3D culture models will improve our basic knowledge on CNS pathology, favoring the identification of novel targets and the definition of the timing and efficacy of current and future therapeutic approaches.

The implementation and standardization of methodologies in basic research is parallel to the development of GMP-scale procedures to produce hiPS-NSCs with reliable NSC signatures and safety profile to be used in gene and cell therapy approaches. The possibility to produce limited numbers of iPSCs from HLA-matched donors or, ultimately, a genetically modified cell product derived from a single “universal donor” (Riolobos et al., 2013; Mattapally et al., 2018; Lanza et al., 2019) that could be easily expanded and cryopreserved with low manufacturing costs would represent a breakthrough in the field of cell therapy for neurodegenerative and demyelinating disease of different origin, including LSDs. However, crucial safety issues must be considered in the perspective of clinical development and application of these strategies in large cohorts of patients. Despite pre-clinical studies demonstrated the absence of engrafted cells expressing pluripotency markers or oncogenic events in mice analyzed 3- and 6-month after being transplanted with hiPS-NSCs (Doerr et al., 2015; Griffin et al., 2015; Meneghini et al., 2017), accurate long-term safety and efficacy assessments (i.e., toxicology, engraftment, long-term persistence) with GMP-scale cell products are required in small and large animals before moving to clinical development. Importantly, guidelines have to be discussed and defined in order to standardize protocols for hiPS-NSC production and transduction, and to define the efficient cell dosage and route of transplantation based on the target area and on the onset, progression and severity of the treated neurodegenerative LSD.

## Author Contributions

ML and VM wrote the manuscript. VM and AG revised the manuscript. VM conceived the review. All the authors contributed to the article and approved the submitted version.

## Conflict of Interest

The authors declare that the research was conducted in the absence of any commercial or financial relationships that could be construed as a potential conflict of interest.
